# Characterization of the Recombinant Exopeptidases PepX and PepN from *Lactobacillus helveticus* ATCC 12046 Important for Food Protein Hydrolysis

**DOI:** 10.1371/journal.pone.0070055

**Published:** 2013-07-19

**Authors:** Timo Stressler, Thomas Eisele, Michael Schlayer, Sabine Lutz-Wahl, Lutz Fischer

**Affiliations:** Department of Biotechnology, Institute of Food Science and Biotechnology, University of Hohenheim, Stuttgart, Germany; Instituto de Tecnologica Química e Biológica, UNL, Portugal

## Abstract

The proline-specific X-prolyl dipeptidyl aminopeptidase (PepX; EC 3.4.14.11) and the general aminopeptidase N (PepN; EC 3.4.11.2) from *Lactobacillus helveticus* ATCC 12046 were produced recombinantly in *E. coli* BL21(DE3) via bioreactor cultivation. The maximum enzymatic activity obtained for PepX was 800 µkat_H-Ala-Pro-*p*NA_ L^−1^, which is approx. 195-fold higher than values published previously. To the best of our knowledge, PepN was expressed in *E. coli* at high levels for the first time. The PepN activity reached 1,000 µkat_H-Ala-*p*NA_ L^−1^. After an automated chromatographic purification, both peptidases were biochemically and kinetically characterized in detail. Substrate inhibition of PepN and product inhibition of both PepX and PepN were discovered for the first time. An apo-enzyme of the Zn^2+^-dependent PepN was generated, which could be reactivated by several metal ions in the order of Co^2+^>Zn^2+^>Mn^2+^>Ca^2+^>Mg^2+^. PepX and PepN exhibited a clear synergistic effect in casein hydrolysis studies. Here, the relative degree of hydrolysis (rDH) was increased by approx. 132%. Due to the remarkable temperature stability at 50°C and the complementary substrate specificities of both peptidases, a future application in food protein hydrolysis might be possible.

## Introduction

Enzymatic protein hydrolysis is a complex process. The synergistic effects of various peptidases are necessary to achieve a reasonable degree of hydrolysis. Enzymatic hydrolysis of food proteins is widely used to increase the value of the final products by improving its nutritional characteristics, adding functional properties and removing inhibitory peptides [1], [2]. Furthermore, hydrolyzed protein formulas reduce or eliminate allergenicity and prevent and/or treat allergic disorders [3]. Proteolytic enzymes are also used to release bioactive peptides, and casein is an example of this principle. Lactic acid bacteria (LAB) are one potential source of these proteolytic enzymes. Based on their extensive proteolytic systems, these auxotrophic microorganisms are perfectly adapted to grow in milk [4], [5]. Several endo- and exopeptidases from lactobacilli have been isolated and have been the focus of biochemical and genetic studies [4], [6]. The synergistic effect of X-prolyl dipeptidyl aminopeptidase (PepX; EC 3.4.14.11) and the general aminopeptidase N (PepN; EC 3.4.11.2) is important to achieve a high degree of hydrolysis. PepN releases amino acids from the N-terminus of peptides. However, hydrolysis is terminated and/or the velocity of hydrolysis is reduced when proline residues are present [7], [8]. This mechanism of proline inhibition is compensated by the action of PepX, which hydrolyzes X-Pro dipeptides from the N-terminus of peptides. Once the prolines are removed, hydrolysis continues via PepN [9]. The native aminopeptidase PepN from *Lactobacillus helveticus* CNRZ 32 [7] and *Lb. helveticus* LHE-511 [10] were partially purified and biochemically characterized; the optimal pH, temperature and thermal stability were determined. However, no kinetic studies have been reported that investigate substrate or product inhibition during food protein hydrolysis. Neither have any inhibition studies utilizing PepX from different *Lb. helveticus* strains [5], [11], [12] or other LAB exopeptidases been reported. Product inhibition for endopeptidases is a known problem that occurs during food protein hydrolyses [13], [14].

We report the production of recombinant PepN and PepX from *Lb. helveticus*. Both recombinant enzymes were purified and biochemically characterized. Kinetic studies were also performed to investigate substrate and product inhibition. The synergistic effects of PepN and PepX in casein hydrolysis were investigated.

## Materials and Methods

### Materials

All chemicals (analytical grade) were obtained from Sigma-Aldrich Chemie GmbH (Schnelldorf, Germany), Carl Roth (Karlsruhe, Germany), AppliChem GmbH (Darmstadt, Germany), or Merck AG (Darmstadt, Germany). Oligonucleotides were purchased from biomers.net GmbH (Ulm, Germany). Molecular biology kits and enzymes were obtained from Qiagen (Hilden, Germany), Fermentas GmbH (St. Leon-Rot, Germany), New England Biolabs GmbH (NEB; Frankfurt am Main, Germany), or Roche (Mannheim, Germany). Chromogenic peptides and other standard peptides were purchased from Bachem AG (Bubendorf, Switzerland). Casein was obtained from Bayerische Milchindustrie eG (Landshut, Germany). The nucleotide sequence analyses were conducted by SRD – Scientific Research and Development GmbH (Bad Homburg, Germany). Chromatographic material (BioFox 40 IDA_low_) for protein purification and the Bioline chromatography system were purchased from Wissenschaftliche Gerätebau Dr. Ing. Herbert Knauer GmbH (Berlin, Germany). The bioreactor cultivations were performed using the Multifors system (Infors AG, Bottmingen/Basel, Switzerland). The MINI-PROTEAN system (Bio-Rad Laboratories GmbH, München, Germany) was used for polyacrylamide gel electrophoresis.

### Bacterial Strains and Culture Conditions

The strain *Lactobacillus helveticus* ATCC 12046 was cultivated in de Man, Rogosa and Sharpe (MRS) medium [15] with constant shaking at 37°C. *E. coli* DH5α (Invitrogen, Carlsbad, USA) and *E. coli* BL21(DE3) (Novagen, Madison, USA) strains were used as hosts for plasmid maintenance and T7 expression work, respectively. Standard protocols were used for the preparation and transformation of competent *E. coli* cells with plasmid DNA via heat shock [16]. Cells were cultivated in Luria Bertani (LB) medium supplemented with the appropriate antibiotic (100 µg mL^−1^ ampicillin) and agar (15 g L^−1^) for agar plates. All cultures were grown with continuous shaking at 37°C unless otherwise stated.

### Cloning, Construction of Expression Vectors and Sequencing of *pepX* and *pepN*

The total genomic DNA from *Lb. helveticus* ATCC 12046 was extracted using an identical method as previously described [17]. Polymerase chain reaction (PCR) was performed using HotStar HiFidelity polymerase (Qiagen), according to the manufacturer’s instructions. The primers *pepX*_for (5′-CGGGATCCATGAAATGAAATATAACCAATATG-3′) and *pepX*_rev (5′-CGGCATGCTTAGGATTAATTATTTTTCATAAAA-3′) were used for the amplification of the *pepX* gene based on the nucleotide sequence of *pepX* from *Lactobacillus helveticus* CNRZ 32 (EMBL: AAB50275) that is available in the UniProt database (UniProt ID: Q59485). The PCR product (approx. 2,400 bp) of *pepX* (2,379 bp) was cloned into the pJET1.2 vector (Fermentas), according to the manufacturer’s instructions. Similarly, the *pepX* gene was amplified with the primers *NdeI*_*pepX*_for (5′-AGAGTCTGCATATGAAATATAACCAATATGC-3′) and *XhoI*_*pepX*_rev (5′-ACTAATCTCGAGTTTTTCATAAAACTTGATTTCA-3′). *NdeI*_*pepN*_for (5′-CAGGCAAACATATGGCAGTTAAACGTTTC-3′) and *XhoI*_*pepN*_rev (5′-ACTAATCTCGAGATCAATTGCTTTAGCAACTGC-3′) primers were used for the *pepN* gene (2,532 bp) based on the nucleotide sequence of the *pepN* gene from *Lactobacillus helveticus* (EMBL: CBK51574) that is available in the UniProt database (UniProt ID: Q10730), resulting in an approx. 2,500 bp PCR product.

The PCR products of *pepX* (pJET1.2 as template) and *pepN* (genomic DNA as template) were purified (QIAquick Gel Extraction Kit; Qiagen) after electrophoresis through an agarose gel (0.8%). The construction of the expression vectors pET-20b(+)_*pepX* and pET-20b(+)_*pepN*, nucleotide sequence analyses and database searches were conducted, as described previously [17].

### Expression of Recombinant PepX and PepN in *E. coli* BL21(DE3)

Transformed *E. coli* BL21(DE3) strains were grown in 2× YT medium that contained glucose (10 g L^−1^) supplemented with ampicillin (100 µg mL^−1^). Precultures were incubated at 37°C on a rotary shaker. The first precultures were cultivated for 18 h and the second precultures for 13 h. The main cultures (800 mL) were grown in a bioreactor parallel system (Multifors), following the analytical methods previously described [17], with some modifications. The stirrer speed varied between 500 and 1000 rpm. The temperature was maintained at 30°C until the OD_600_ reached a value of 5 to minimize the formation of inclusion bodies, and protein expression was induced by the addition of 0.5 mM IPTG. During the cultivations, samples were removed at various time points, and the enzymatic activity was determined from the cell-free extract after cell disruption [17]. The cultures were harvested after 23 h of cultivation, as previously described [17].

### Automated Purification of PepX and PepN

Both PepX and PepN were individually purified using Ni^2+^ immobilized metal affinity chromatography (IMAC) and subsequently desalted via two HiPrep™ 26/10 columns using an automated operating procedure, as previously reported [17], [18]. Cell suspensions of 15% (w/v) were prepared in 50 mM Na_2_HPO_4_/KH_2_PO_4_ buffer (pH 6.5) containing 500 mM NaCl and 20 mM imidazole (PepX) or 10 mM imidazole (PepN). Both enzymes were eluted by increasing the imidazole concentration to 500 mM in an identical buffer. Subsequently, the enzymes were desalted in 50 mM Na_2_HPO_4_/KH_2_PO_4_ buffer (pH 6.5).

### Polyacrylamide Gel Electrophoresis (PAGE)

The samples, following cell disruption (sonication), were divided into soluble and insoluble fractions. These samples and purified PepX and PepN (5 µg of protein each; [19]) were analyzed by sodium dodecyl sulfate (SDS) PAGE (12.5% gel) [20]. A standard molecular weight protein mixture was used as a reference (NEB). Gels were stained with Coomassie Brilliant Blue to detect the proteins.

Native PAGE (8% gel) was conducted on ice (4°C) with soluble samples following purification (5 µg of protein each; [19]). A native standard molecular weight protein mixture was obtained from SERVA Electrophoresis GmbH (Heidelberg, Germany) and was used as a reference. Gels were stained with Coomassie Brilliant Blue to detect the proteins. A solution was prepared for activity staining containing H-Ala-Pro-*p*NA (PepX) or H-Ala-*p*NA (PepN) (5 mg of each) dissolved in dimethylformamide (DMF; 250 µL) and diluted to a final volume of 10 mL with Na_2_HPO_4_/KH_2_PO_4_ buffer (50 mM; pH 6.5). The lanes of the native PAGE gel used for activity staining were excised and then incubated with the corresponding *p*NA solution at 37°C until yellow bands were observed. The yellow bands developed were redyed to violet-colored bands using the following protocol [21]. The lanes of the native PAGE gel containing yellow bands were incubated in a 0.1% (w/v) NaNO_2_/1 M HCl solution until the bands disappeared (approx. 2 min). The lanes were then washed with 1% (w/v) urea/H_2_O_dd_ for 30 sec. Finally, the bands were developed by incubating the native PAGE lanes in 0.025% (w/v) N-1-naphthyl-ethylenediamine dihydrochloride/22% (v/v) EtOH for approx. 10 min, allowing the violet color to appear. The native PAGE gel was digitally imaged before the violet color disappeared (approx. 5–30 min).

### Measurement of Proteolytic Enzymatic Activity Using Chromogenic Peptides

The chromogenic peptides H-Ala-Pro-*p*NA and H-Ala-*p*NA were used to determine the proteolytic activities of PepX and PepN, respectively, in a standard assay. The standard assay was performed as follows. Initially, 50.5 µL of the enzyme solution was added to 177 µL of Na_2_HPO_4_/KH_2_PO_4_ buffer (50 mM; pH 6.5). After incubation at 37°C for 10 min, 12.5 µL of the chromogenic peptide solution (5 mg mL^−1^ for PepX; 7.5 mg mL^−1^ for PepN, dissolved in DMF) was added to the reaction mixture. The reaction was terminated by adding 50 µL of 50% (v/v) acetic acid. After centrifugation (8,000×*g*, 5 min), 240 µL of the solution was transferred to a microtiter plate, and the absorption was measured in an iEMS Reader (Labsystems, Helsinki, Finland) at 405 nm. One katal of proteolytic activity was defined as the amount of enzyme required to release 1 mol of *p*-nitroaniline per second.

### Characterization of PepX and PepN

The purified PepX and PepN (see above) were characterized. All experiments were repeated at least once, and the data reported were measured in triplicate. The error bars were not included in the graphical representations if the standard deviation was below 5%.

#### Determination of enzyme kinetics

The kinetic parameters were determined using H-Ala-Pro-pNA, H-Arg-Pro-pNA and H-Gly-Pro-pNA · p-tosylate as the substrates for PepX. The substrates H-Arg-pNA · 2HCl, H-Lys-pNA · 2HBr, H-Leu-pNA, H-Ala-pNA, H-Phe-pNA, H-Val-pNA, H-Ile-pNA, H-Gly-pNA, H-Pro-pNA, H-His-pNA, and H-Glu-pNA were used for PepN. Standard enzymatic activity assay conditions were used in which the substrate concentrations ranged from 0.017 to 7.47 mM, depending on the particular enzyme and substrate. The enzyme solution was preincubated with the inhibitor at 37°C for 15 min for product inhibition kinetic studies.

#### Effect of pH and temperature on the enzymatic activity

The activity was assayed under standard conditions in which the enzymes were diluted and the assay buffer was replaced by the corresponding buffer to determine the pH profiles of PepX and PepN. The pH values ranged from 5 to 10.

The activities were assayed under standard conditions in which the assay temperatures ranged from 10 to 65°C (PepX) or from 10 to 55°C (PepN) to assess the effect of temperature on the enzymatic activity. The enzyme solutions were incubated at different temperatures (0 to 50°C) for 15 d to determine the thermal stability. Subsequently, the residual activity was measured under standard conditions (see above).

#### Effect of inhibitors, cations, metal chelators, reducing agents, and solvents on the enzymatic activity

The substances tested were solubilized and diluted in H_2_O_dd_, dimethyl sulfoxide (DMSO), acetone, or ethanol, depending on the substance. The assay conditions were identical to the standard protocol, except that 24 µL of the test substance and 153 µL of buffer were used, instead of 177 µL of buffer. All salt and metal ions were added as chlorides to prevent any influence of anions.

Apo-PepN was prepared by treating active and native PepN with 10 mM ethylenediaminetetraacetic acid (EDTA) followed by dialysis in 50 mM Na_2_HPO_4_/KH_2_PO_4_ buffer (pH 6.5). The PepN treated with EDTA exhibited no activity. However, the activity was restored after dialysis. We conclude that the divalent cations, which were dissolved in H_2_O_dd_, were adequate to restore the PepN activity. No PepN activity was detectable after the addition of 0.03 mM EDTA to the dialyzed enzyme preparation and the enzyme solution was used for recovery experiments.

### Hydrolysis of Casein with Alcalase, PepX and PepN

The prehydrolysis of casein was performed using Alcalase 2.4 L (Novozymes, Bagsvaerd, Denmark). The improved hydrolyses were performed with purified PepX and PepN. All experiments were repeated at least once, and the data reported were measured in triplicate. The error bars were not included in the graphical representations because the standard deviation was below 5%.

#### Determination of the proteolytic enzymatic activity of alcalase with o-phthaldialdehyde (OPA)

The initial enzymatic activity of Alcalase 2.4 L was determined using casein (1%, w/v) in Na_2_HPO_4_/KH_2_PO_4_ buffer (50 mM; pH 7.0) as the substrate. The reaction contained 250 µL of the substrate and 25 µL of diluted Alcalase 2.4 L solution, was incubated at 37°C and terminated at various time points with the addition of 25 µL trichloroacetic acid (TCA; 2 M). Following centrifugation (8,000×*g*, 5 min), 25 µL of the solution was transferred to a microtiter plate, and 175 µL of the OPA reagent [22] was added while shaking for 5 sec. After exactly 2 min, the absorption was measured in an iEMS Reader (Labsystems, Helsinki, Finland) at 340 nm. The respective sample was mixed with TCA before adding the substrate for reference. One katal of proteolytic activity was defined as the amount of enzyme required to release 1 mol of serine equivalents per second.

#### Prehydrolysis of casein with alcalase

Casein (2.5%, w/v; 100 mL) was suspended in Na_2_HPO_4_/KH_2_PO_4_ buffer (50 mM; pH 7.0), and sodium azide (0.1%, w/v) was added. Alcalase (480 nkat mL_solution_^−1^) was added after preincubation of the solution (37°C, 30 min). Samples (450 µL) were removed at various time points during hydrolysis until no increase in serine equivalents was observed (6.5 h). The samples were added to 50 µL of SDS (10%, w/v) and heated to 80°C for 10 min for enzyme inactivation. After centrifugation (8,000×*g*, 5 min), the samples were analyzed using the OPA assay described above. The inactivated samples were stored at −20°C for analyses conducted later. The hydrolyzed casein solution was heated to 75°C for 30 min and stored at −20°C for improved hydrolysis measurement.

#### Improved hydrolysis with PepX and PepN

The prehydrolyzed casein solution (see above) was used for improved casein hydrolysis (10 mL). Sodium azide (0.1%, w/v) was added to the prehydrolyzed casein solution and incubated at 37°C for 30 min. PepX (50 nkat mL_solution_^−1^), PepN (50 nkat mL_solution_^−1^) or both enzymes (each 50 nkat mL_solution_^−1^) were added for further hydrolysis (10 h). Samples were removed at various time points during hydrolysis and were treated as described above.

## Results

The original wild-type strain produced only low levels of PepX and PepN when cultivated in typical MRS medium (data not shown). Therefore, an efficient heterologous recombinant production system was established in *E. coli* to obtain high levels of both enzymes for more detailed biochemical and kinetic characterization.

### Sequencing of *pepX* and *pepN* from *Lactobacillus helveticus* ATCC 12046

The PepX expression vector (pET-20b(+)_*pepX*) was constructed, sequenced (Sequence S1) and used for expression in the *E. coli* BL21(DE3) strain. The nucleotide sequence of the *pepX* gene obtained was deposited in the GenBank database (accession number: JX682666) and exhibited 100% identity to that of *Lb. helveticus* CNRZ 32.

The PepN expression vector (pET-20b(+)_*pepN*) was also constructed and sequenced (Sequence S2), and the gene sequence for *pepN* obtained was deposited in the GenBank database (accession number: JX682667). PepN exhibited 99% sequence identity to the corresponding gene of *Lb. helveticus* CNRZ 32.

### Heterologous Expression of PepX and PepN in *E. coli*

**Figure 1:**
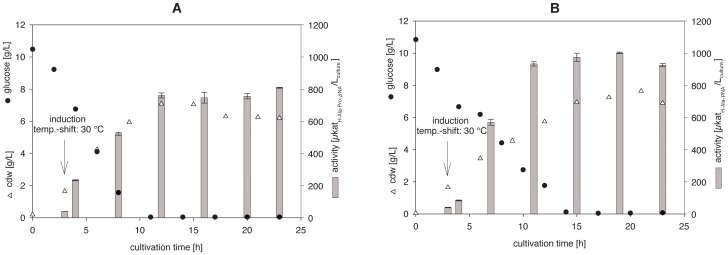
Recombinant production of PepX (A) and PepN (B) from *Lb.* *helveticus* ATCC 12046 expressed in *E. coli* BL21(DE3). Cultivation began at 37°C and shifted to 30°C with simultaneous induction using IPTG (see arrow). The glucose consumption, biomass and particulate enzyme production is presented. (Bioreactor working volume: 800 mL; each point represents the average of triplicate measurements).

High levels of soluble PepX or PepN were individually expressed in *E. coli* BL21(DE3) using identical cultivation conditions (Fig. 1).

During the cultivation of the recombinant pET-20b(+)_*pepX* in *E. coli* BL21(DE3), the glucose was completely consumed after approx. 11 h when the cells entered the stationary phase (Fig. 1A). The OD_600 nm_ value for the cells increased up to 26 during cultivation (data not shown), and the maximum enzymatic activity of PepX was 800 µkat_H-Ala-Pro-*p*NA_ L^−1^. The enzymatic activity was approximately constant over 11 h during the stationary phase. The wet cell biomass corresponded to 21 g L^−1^ (equal to approx. 6.5 g_cdw_ L^−1^).

An OD_600 nm_ value of 33 was observed for the production of recombinant PepN for the *E. coli* during cultivation (data not shown). The glucose was completely consumed by the cells after 14.5 h when entering the stationary phase (Fig. 1B). The maximum enzymatic activity of PepN was determined to be 1,000 µkat_H-Ala-*p*NA_ L^−1^, which was nearly constant during the stationary phase. The wet cell biomass concentration at the end of the cultivation was 27 g L^−1^ (equal to approx. 8 g_cdw_ L^−1^).

Cells transformed with insert-free pET-20b(+) (reference) were cultivated under identical conditions to determine the background peptidase activities of the *E. coli* BL21(DE) strain. The background activities of the reference were approx. 0.025% for PepX and 1% for PepN.

### Automated Purification of Recombinant PepX and PepN

Both His-tagged PepX and PepN were individually purified using an automated chromatographic procedure based on Ni-IMAC (see Figure S1 for the chromatogram). An enzymatic activity yield of 93% and a purification factor of 3.7 (specific activity: 967 nkat_H-Ala-Pro-*p*NA_ mg_protein_^−1^) were obtained for PepX. The enzymatic activity yield for PepN was 78% after purification, with a purification factor of 2.9 (specific activity: 577 nkat_H-Ala-*p*NA_ mg_protein_^−1^).

### Molecular Mass Estimations

The SDS PAGE analysis of the peptidase samples, which were obtained from the cultures, indicated overexpressed protein bands at approx. 90 kDa for PepX and 95 kDa for PepN (data not shown). The SDS PAGE analyses of both purified enzymes after affinity chromatography and desalting are shown in Figure S2.

Native PAGE of the purified enzyme fractions, which were stained with Coomassie Brilliant Blue, and also for peptidase activity using the chromogenic substrates, resulted in single protein bands at approx. 180 kDa for PepX and 95 kDa for PepN (see Figure S2). These results suggest that the purified PepX was a homodimer with an apparent molecular mass of approx. 180 kDa, for which the theoretical calculation is 183.1 kDa (based on the amino acid sequence plus the His-tag). The purified PepN was a monomer with an apparent molecular mass of 95 kDa, for which the theoretical calculation is 96.8 kDa (based on the amino acid sequence plus the His-tag).

### Characterization of PepX

#### pH and temperature optimum and thermal stability

**Figure 2:**
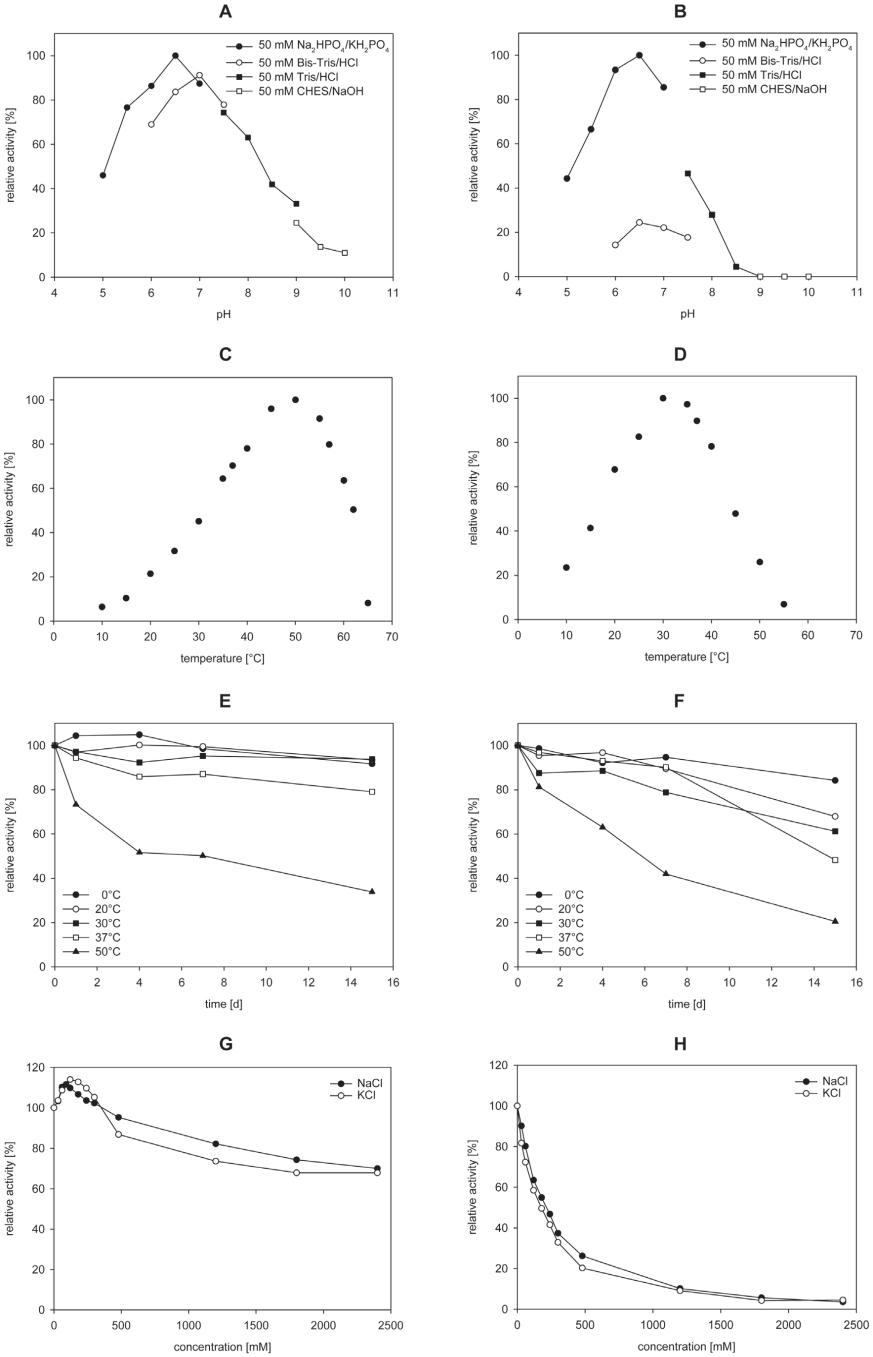
Characterization of the purified PepX (A; C; E; G) and PepN (B; D; F; H) from *Lb.* *helveticus* ATCC 12046. A and B: pH optimum; C and D: temperature optimum; E and F: thermal stability; G and H: influence of NaCl and KCl (each point represents the average of triplicate measurements; the standard deviation was <5%).

PepX exhibited the highest activity in Na_2_HPO_4_/KH_2_PO_4_ buffer at pH 6.5 (100%; Fig. 2A) whereas it exhibited higher activity in Bis-Tris/HCl buffer at pH 7.0 (91%) than at pH 6.5 (84%). The activity in Na_2_HPO_4_/KH_2_PO_4_ buffer at pH 7.0 was only 87%. The highest activity of PepX was obtained at 50°C (100%; Fig. 2C), and it demonstrated a residual activity of only 8% at 65°C. PepX was preincubated at various temperatures over a period of 15 d to determine the thermal stability (Fig. 2E). The greatest thermal stability for the enzyme was observed at 0, 20 and 30°C, which resulted in more than 90% of the residual activity. Even at temperatures of 37 and 50°C, PepX exhibited residual activities of 79 and 34%, respectively. These results demonstrated the high thermal stability of this enzyme and its potential for application in industrial hydrolyses processes.

#### Kinetic studies

The kinetic experiments for PepX were conducted with the commercially available chromogenic substrates H-Ala-Pro-*p*NA, H-Arg-Pro-*p*NA and H-Gly-Pro-*p*NA·*p*-tosylate (Table 1). No substrate inhibition was observed in the concentration range tested (0.017–4.26 mM). The corresponding *K_m_* and *V_max_* values were calculated from Hanes plots. The *K_m_* values determined were in the range between 1.3 and 11.3 mM. The highest relative catalytic efficiency (*V_max_*/*K_m_*) for PepX was obtained with the substrate H-Ala-Pro-*p*NA (100%), followed by H-Arg-Pro-*p*NA (59%) and H-Gly-Pro-*p*NA·*p*-tosylate (6%).

**Table 1:** Kinetic parameters of PepX^1^ using chromogenic *p*-nitroanilide substrates.

			Catalytic efficiency	
Substrate	*V_max_* [nkat mL^−1^]	*K_m_* [mM]	*V_max_/K_m_* [1 s^−1^]	relative [%]
H-Ala-Pro-*p*NA (reference)^2^	2309	1.3	1.76	100
H-Arg-Pro-*p*NA	1549	1.5	1.04	59
H-Gly-Pro-*p*NA·*p*-tosylate	1224	11.3	0.11	6

1 Protein concentration of the enzyme solution: 2.61 mg mL^−1^.

2 Substrate for the standard assay. Triplicate measurements; the standard deviation was <5%.

**Figure 3:**
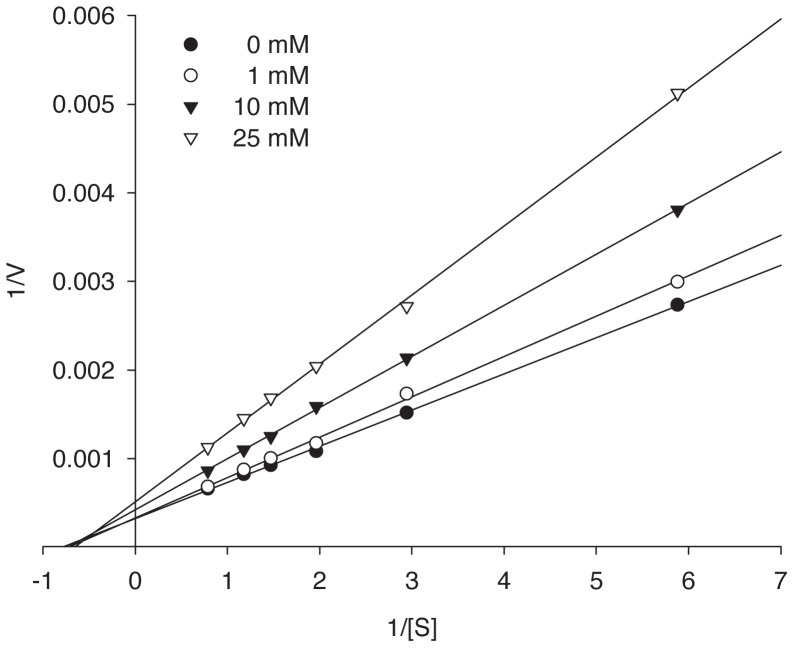
Lineweaver-Burk linearization of PepX inhibition by H-L-Phe-L-Pro-OH (H-Ala-Pro-*p*NA as substrate; each point represents the average of triplicate measurements; the standard deviation was <5%).

In addition, the potential inhibitory influence of PepX products (H-X-Pro-OH peptides) on the initial PepX activity was investigated. Therefore, the potential PepX products H-Gly-L-Pro-OH, H-L-Ala-L-Pro-OH, H-L-Arg-L-Pro-OH, H-L-Pro-L-Pro-OH, H-L-Phe-L-Pro-OH, and H-L-Tyr-L-Pro-OH were added to the assays at final concentrations of 0.1, 1 or 10 mM. Inhibition was detected for the peptides containing an aromatic amino acid in their sequence. PepX was inhibited by 28% when H-L-Tyr-L-Pro-OH was present at a concentration of 10 mM. An even stronger inhibition of 38% was observed in the presence of 10 mM H-L-Phe-L-Pro-OH. A representative product inhibition is shown in Figure 3. Lineweaver-Burk linearization indicated that the inhibition was mixed-type (coincident noncompetitive and competitive inhibition). The inhibition constants were 30.1 mM for *K_i_*, which is the binding constant for the enzyme, and 43.6 mM for *K_i_ ´*, which is the binding constant for the enzyme-substrate complex.

#### Effect of salts and metal ions on PepX activity

The influence of different mono- and divalent cations on PepX activity was extensively tested to characterize its behavior in a complex environment, such as food (Fig. 2G, Table 2). Concentrations of up to 300 mM of NaCl and KCl exerted a favorable effect on PepX activity (Fig. 2G). Conversely, PepX was inhibited by most divalent cations (Table 2). Nearly complete inhibition of the enzymatic activity was observed in the presence of 10 mM CuCl_2_ or 0.0001 mM HgCl_2_. No inhibition at low ZnCl_2_ concentrations (0.1 and 1 mM) was observed. No inhibition for the monovalent cation NH_4_^+^ (NH_4_Cl) was observed up to a concentration of 10 mM.

**Table 2:** Effect of various solvents, cations, inhibitors, reducing agents, metal chelators, and denaturing agents on the activity of PepX and PepN from *Lb. helveticus* ATCC 12046 (at 37°C in 50 mM Na_2_HPO_4_/KH_2_PO_4_ buffer, pH 6.5).

			Activity [%]^1^				Activity [%]^1^	
	Substance	Concentration	PepX	PepN	Substance	Concentration	PepX	PepN
**Solvents [% (v/v)]**	Acetone	10	50	13	DMSO	10	74	102
	EtOH	10	78	18	DMF	10	37	23
**Cations^2^ [mM]**	Ca^2+^	0.1	99	99	Mg^2+^	0.1	90	96
		1	89	65		1	89	92
		10	59	8		10	90	58
	Co^2+^	0.1	99	186	Mn^2+^	0.1	94	76
		1	90	161		1	92	36
		10	70	55		10	59	10
	Cu^2+^	0.001	96	54	NH_4_^+^	0.1	99	95
		0.01	89	39		1	99	96
		0.1	34	20		10	100	94
		1	21	2	Sn^2+^	0.1	93	89
		10	1	0		1	95	82
	Fe^2+^	0.1	91	79		10	20	21
		1	58	6	Zn^2+^	0.1	125	78
		10	37	4		1	137	34
	Hg^2+^	0.0001	6	94		10	36	13
		0.001	3	2				
		0.01	0.2	0				
**Reagents [mM]**	*β*-mercaptoethanol^2^	0.1	108	74	Pepstatin A^3^	0.001	97	95
		1	104	64		0.01	92	90
		10	105	39		0.1	87	86
	DTT^2^	0.1	100	110	1,10-phenanthroline^4^	0.001	n.d.	102
		1	106	86		0.01	n.d.	21
		10	105	29		0.1	100	0
	EDTA^2^	0.000001	n.d.	86		1	100	0
		0.00001	n.d.	75		10	100	0
		0.0001	n.d.	44	PMSF^5^	0.1	96	102
		0.001	n.d.	8		1	66	85
		0.01	n.d.	1		10	11	74
		0.1	104	0	SDS^2^	0.1	106	80
		1	104	0		1	84	0
		10	113	0		10	0	0
	Imidazole^2^	0.1	97	84	Urea^2^	0.1	101	99
		1	94	86		1	100	94
		10	92	87		10	102	90

1 The value of 100% was determined in the presence of the corresponding solvent without the additional substance. The substance was dissolved in: ^2^H_2_O_dd_; ^3^DMSO; ^4^Acetone; ^5^EtOH. Triplicate measurements; the standard deviation was <5%; n.d., not determined.

#### Effect of organic solvents, peptidase inhibitors, reducing agents, metal chelators, and denaturing agents on PepX activity

The substrates and the peptidase inhibitors were dissolved in organic solvents prior to their addition to the assay due to their limited solubility in pure water. The standard PepX assay, which used H-Ala-Pro-*p*NA as the substrate, contained 5.2% (v/v) DMF. In order to determine the individual influence of each organic solvent on PepX, the standard activity was measured in the presence of an additional 10% (v/v) of the particular solvent (Table 2). Additional DMF decreased the PepX activity to 37%. The addition of acetone, DMSO and ethanol resulted in 50, 74 and 78% residual activity, respectively. Thus, either DMSO or ethanol would be more suitable organic solvents than DMF for PepX. However, DMF is a suitable organic solvent for substrates that are not water soluble.

The enzymatic activities measured in the presence of an additional 10% (v/v) of acetone, DMSO and ethanol were considered 100% for the inhibition studies using 1,10-phenanthroline, Pepstatin A and phenylmethylsulfonyl fluoride (PMSF), respectively. PMSF (10 mM), a specific serine peptidase inhibitor, reduced PepX activity the most to 11% residual activity. Additionally, inhibition was observed using 0.1 mM Pepstatin A, which is a specific carboxy peptidase inhibitor. However, higher concentrations could not be investigated due to the limited solubility of this inhibitor.

Reducing agents such as DTT (dithiothreitol) and *β*-mercaptoethanol at concentrations up to 10 mM did not inactivate PepX. This result indicates that potential disulfide groups do not appear to be essential for the enzymatic activity [5]. No inhibition was observed with metal-complexing reagents, such as EDTA and 1,10-phenanthroline (10 mM). A complete inactivation of PepX was observed in the presence of the anionic surfactant SDS (10 mM), as expected.

### Characterization of PepN

#### pH and temperature optimum and thermal stability

The pH and temperature optimum of the purified PepN was investigated (Figs. 2B, 2D, 2F). PepN exhibited the highest activity in Na_2_HPO_4_/KH_2_PO_4_ buffer at pH 6.5 (100%). Bis-Tris/HCl buffer demonstrated a strong negative effect on the enzymatic activity (24% at pH 6.5; Fig. 2B). The reason for this effect is not clear.

Concerning the optimum assay temperature for PepN, the highest activity was observed at an unusually low temperature of 30°C (100%; Fig. 2D). A residual PepN activity of only 26% was measured at an assay temperature of 50°C. Concerning the thermal stability, incubation for 15 d resulted in residual PepN activities of 84% at 0°C, 61% at 30°C and 20% at 50°C (Fig. 2F). It is remarkable that PepN was not completely inactivated after incubation at 50°C for 15 d because of its low temperature optimum of 30°C. The inactivating effect of DMF, which was present in the standard assay used in the measurements of the optimal temperature, might explain the low optimal temperature. This result is consistent with the results below (see Table 2) in which a strong PepN sensitivity to DMF was observed (the addition of 10% (v/v) DMF resulted in 23% residual activity).

#### Kinetic studies

**Figure 4:**
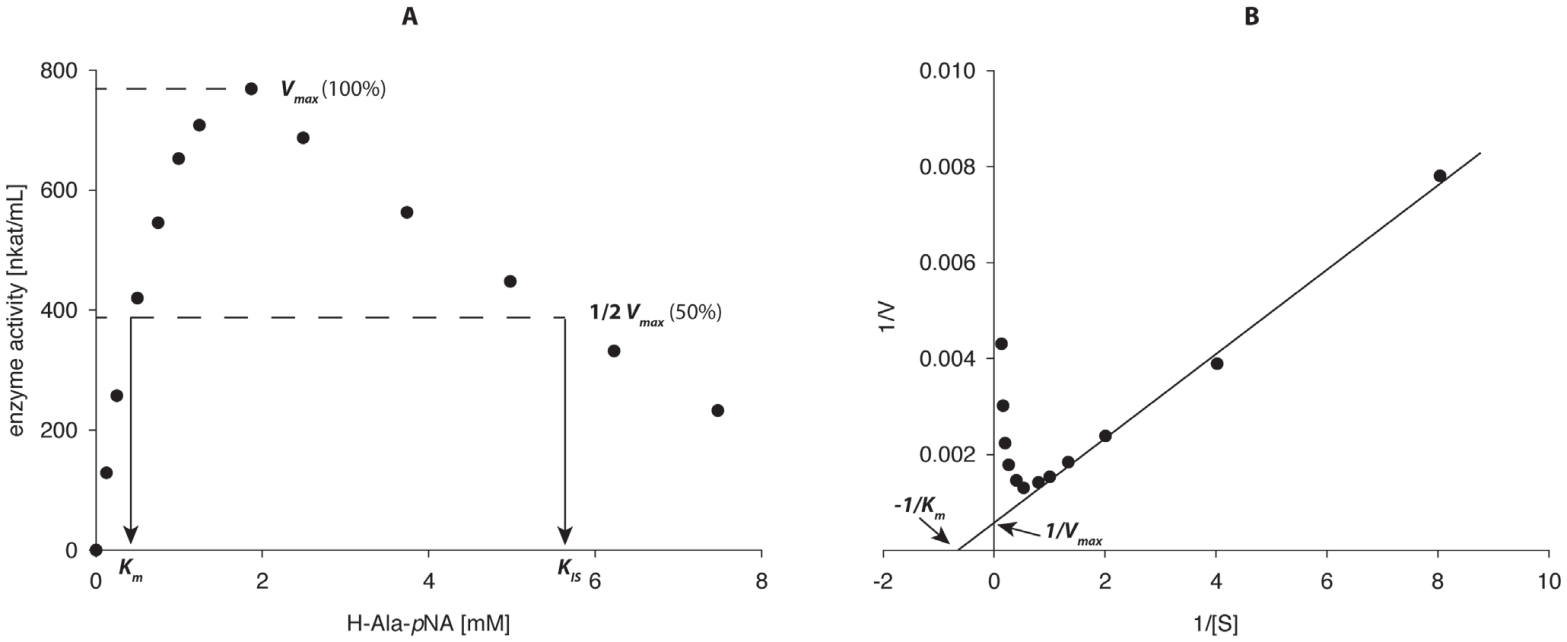
Determination of the kinetic parameters of PepN for the substrate H-Ala-*p*NA according to Michaelis-Menten analysis (A) and Lineweaver-Burk linearization (B) (each point represents the average of triplicate measurements; the standard deviation was <5%).

The kinetic parameters for PepN using different chromogenic substrates are summarized in Table 3. PepN demonstrated broad substrate specificity, which included hydrophobic, aromatic, acidic, and basic amino acids. A PepN peptidase activity was detected for H-Glu-*p*NA and H-His-*p*NA (data not shown), but due to the poor solubility properties of these substrates, a determination of the kinetic constants was not possible. The kinetic parameters of the soluble substrates were determined by both Michaelis-Menten and Lineweaver-Burk analyses, because all substrates demonstrated clear substrate inhibition at higher concentrations. Representative kinetic analyses obtained for H-Ala-*p*NA (used as a substrate in the standard assay) are presented in Figure 4. The values for *V_max_* and *K_m_* determined by Lineweaver-Burk analysis were always higher than those determined by Michaelis-Menten analysis, due to the linearization and the substrate inhibition. However, Michaelis-Menten analysis was more suitable for determining the inhibition constants (*K_IS_)* for the enzyme (the substrate concentration at half *V_max_* inhibition).

**Table 3:** Kinetic parameters of PepN^1^ using chromogenic *p*-nitroanilide substrates.

	Michaelis-Menten			Lineweaver-Burk			
						Catalytic efficiency	
Substrate	V_max_ [nkat mL^−1^]	K_m_ [mM]	K_IS_ [mM]	V_max_ [nkat mL^−1^]	K_m_ [mM]	V_max_/K_m_ [1 s^−1^]	relative [%]
H-Ala-pNA (reference)^2^	769	0.43	5.72	1619	1.43	1.13	9.91
H-Lys-pNA · 2HBr	1552	0.14	9.91	2068	0.18	11.40	100
H-Arg-pNA · 2HCl	1742	0.14	4.98	3619	0.42	8.62	75.6
H-Leu-pNA	851	0.17	2.92	1240	0.29	4.28	37.5
H-Phe-pNA^3^	254	0.15	1.87	385	0.24	1.60	14.0
H-Val-pNA	30.7	0.27	3.41	49.8	0.63	0.08	0.70
H-Ile-pNA	10.1	0.17	2.17	17.4	0.38	0.05	0.44
H-Pro-pNA	19.9	0.94	7.15	53.5	3.71	0.01	0.13
H-Gly-pNA	15.8	0.69	8.87	37.8	2.70	0.01	0.13

1 Protein concentration of the enzyme solution: 2.16 mg mL^−1^.

2 Substrate for the standard assay.

3 The maximum substrate solubility was 3.73 mM under the assay conditions. Triplicate measurements; the standard deviation was <5%.

High *V_max_* values were measured using the substrates H-Lys-*p*NA and H-Arg-*p*NA followed by H-Ala-*p*NA and H-Leu-*p*NA. The lowest *K_m_* value was obtained using the substrate H-Lys-*p*NA, which also led to the best hydrolytic efficiency (*V_max_*/*K_m_*) of 11.4 s^−1^. This is the first time, to the best of our knowledge, that substrate inhibition has been clearly observed for PepN. The *K_IS_* values were approx 7.6-fold (H-Pro-*p*NA) to 70-fold (H-Lys-*p*NA) of the corresponding *K_m_* values.

A selection of various free amino acids were added to the standard assay to additionally determine the potential product inhibition of PepN (Table 4). Significant product inhibition was observed using the amino acids L-Phe (10 mM; 24% residual activity) and L-Arg (10 mM; 19% residual activity), only a slight inhibition was observed for L-Ala and Gly, and L-Pro and L-Asp did not inhibit PepN within the concentration range tested. Therefore, product inhibition was not as general as substrate inhibition, but clearly occurred in some cases.

**Table 4:** Relative PepN activities (%) for the standard assay (substrate: H-Ala-*p*NA) in the presence of various potential product inhibitors.

	Concentration [mM]		
Potential product inhibitors	0.1	1	10
L-Ala	96	92	89
L-Arg	93	65	19
L-Asp	99	99	97
Gly	98	96	89
L-Phe	98	75	24
L-Pro	100	101	103

Triplicate measurements; the standard deviation was <3%.

#### Effect of salts and metal ions on PepN activity

PepN activity was also measured in the presence of salts and metal ions to gain insights into a potential industrial application in complex matrices. PepN activity decreased at low NaCl or KCl concentrations (Fig. 2H) and was also inhibited by several divalent cations (Table 2). Here, PepN was strongly inhibited by Cu^2+^ (1 mM; 2% residual activity), Fe^2+^ (1 mM; 6% residual activity) and Hg^2+^ (0.001 mM; 2% residual activity). Almost no inhibition was observed for the monovalent cation NH_4_^+^ (NH_4_Cl) at concentrations up to 10 mM. The divalent cation Co^2+^ demonstrated an activating effect on PepN activity (186–161%) at lower concentrations (0.1–1 mM).

**Figure 5:**
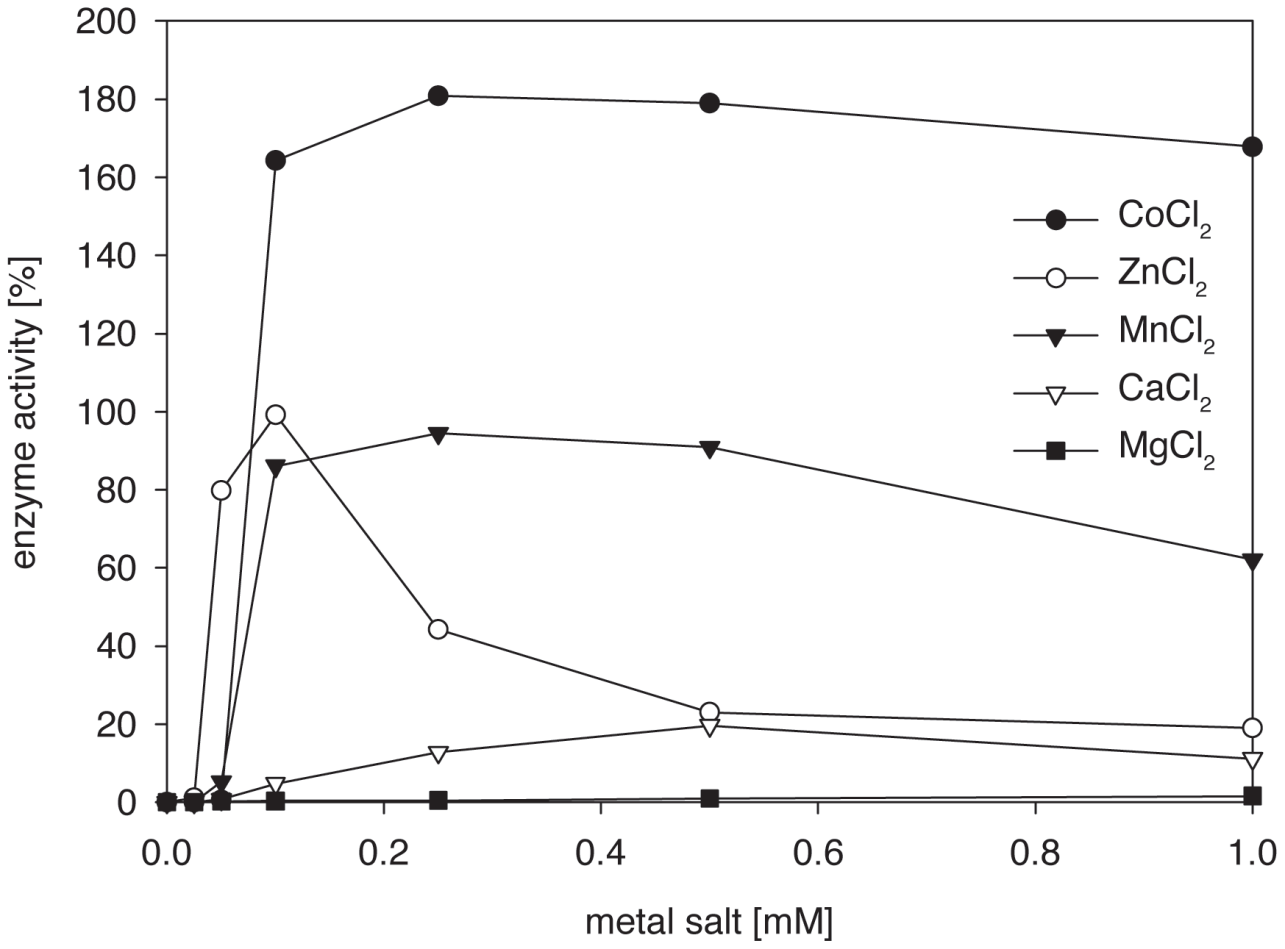
Activation effects of various divalent metal ions on apo-PepN. The enzymatic activity of the native PepN was considered 100% (each point represents the average of triplicate measurements; the standard deviation was <5%).

PepN is known to be a neutral zinc metallopeptidase [23], [24]. We prepared the apo-enzyme (apo-PepN) and investigated the reactivating effect of divalent metal ions. Apo-PepN was incubated with different concentrations of divalent cations (Fig. 5). Co^2+^ was found to enhance PepN activity to 180% at a concentration of 0.25 mM CoCl_2_, And even higher concentrations of the latter, up to 1 mM, preserved this high activity. The “natural” Zn^2+^ ion at a concentration of 0.1 mM restored the PepN activity to 99% compared with the activity of native holo-PepN. However, higher concentrations of ZnCl_2_ decreased the PepN activity, which was consistent with the inhibition studies (Table 2). Only slightly lower recovery yields of up to 94% were obtained with MnCl_2_ in the broad range of 0.1–1 mM.

These results suggest that a replacement of the native Zn^2+^ ion of PepN by a Co^2+^ ion would be useful for a potential biotechnological application of PepN due to the significant increase of its catalytic power.

#### Effects of organic solvents, peptidase inhibitors, reducing agents, metal chelators, and denaturing agents on the enzymatic activity

Similar to PepX, the influence of various agents on the enzymatic activity of PepN were investigated. The results are summarized in Table 2 and similar calculations were performed, as previously described. The enzymatic activity of PepN was strongly reduced by the addition of 10% (v/v) acetone, ethanol and DMF to 13–23% of the reference activity. It was surprising that the addition of DMSO did not influence the PepN activity (102%). An insignificant inhibition was observed using the specific carboxy peptidase inhibitor Pepstatin A (86% residual activity at 0.1 mM) and the serine peptidase inhibitor PMSF (74% residual activity at 10 mM). A complete inactivation of PepN was observed with low concentrations (0.1 mM) of metal-complexing agents, such as EDTA and 1,10-phenanthroline, indicating that PepN is a metalloenzyme, as previously reported.

The inactivation of PepN caused by S-S reducing agents, such as DTT and *β*-mercaptoethanol, probably indicates that disulfide bonds are essential for the activity of this enzyme. As expected, the anionic surfactant SDS (1 mM) completely inactivated PepN.

### Improved Casein Hydrolysis Using PepX and PepN

**Figure 6:**
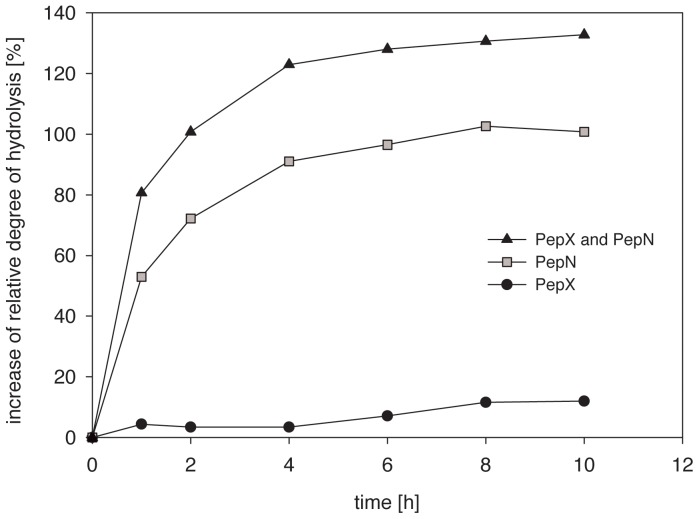
Increase of the relative degree of hydrolysis of a prehydrolyzed casein solution by PepX and/or PepN (each point represents the average of triplicate measurements; the standard deviation was <5%).

The performance of both purified enzymes (PepX, PepN) in milk protein hydrolysis was investigated as follows. Solubilized casein was prehydrolyzed with the commercial endopeptidase preparation Alcalase 2.4 L (480 nkat mL_solution_^−1^) at 37°C over a period of 6.5 h (data not shown). The Alcalase was then inactivated by heat treatment prior to the assay. This prehydrolysis step was necessary because PepX and PepN are exopeptidases, as previously mentioned. PepX and PepN were added individually or in combination to the prehydrolyzed samples (Fig. 6). The highest increase in the rDH was obtained using a combination of PepX and PepN (approx. 132%), which demonstrates the synergistic action of both lactobacilli peptidases. PepX alone increased the rDH only very slightly (approx. 12%) due to its limited substrate specificity (cleavage of X-Pro-peptides at the N-terminus, where X is any amino acid). PepN alone strongly increased the rDH (approx. 100%) due to its very broad substrate specificity, in which all amino acids can be cleaved from the N-terminus until X-Pro-sequences are encountered. In conclusion, the combination of both peptidases provided optimal hydrolysis because of their complementary substrate specificities.

## Discussion

In the current study, PepX (X-prolyl dipeptidyl aminopeptidase; EC 3.4.14.11) and PepN (aminopeptidase N; EC 3.4.11.2) from *Lactobacillus helveticus* ATCC 12046 were cloned and expressed in *E. coli.* The enzymes were purified, characterized and applied in casein hydrolysis.

### 

#### Recombinant production of PepX and PepN

The maximum enzymatic activity of PepX was 800 µkat_H-Ala-Pro-*p*NA_ L^−1^ in the current study. Vesanto and colleagues [25] obtained a PepX activity of only 4.1 µkat_H-Gly-Pro-*p*NA_ L^−1^ for the recombinant PepX from *Lb. helveticus* 53/7; they used the *E. coli* JM105 strain for expression and pKK223-3 as the expression vector, which are not as suitable for the production of the enzyme as our host strain. An activity of 1,000 µkat_H-Ala-*p*NA_ L^−1^ was determined for the recombinant PepN. The expression of PepN from *Lb. helveticus* WSU19 using *E. coli* DH5α has been published previously [26]. However, the authors did not report the enzymatic activity obtained. The recombinant expression of PepN was also obtained in *Lactococcus lactis* [27], but comparable activity data were not provided by the authors.

#### Molecular mass estimation of PepX and PepN

The molecular masses of PepX and PepN were estimated by PAGE after automated purification. PepX from *Lb. helveticus* ATCC 12046 appeared as a homodimer on native PAGE with an estimated molecular mass of 180 kDa. Dimeric forms of PepX were also described from *Lb. helveticus* 57/3 [25] and IFO3809 [28]. In contrast, monomeric PepX from *Lb. helveticus* ITG LH1 [5] and CNRZ 32 [11], [29] have also been reported previously. The PepN from *Lb. helveticus* ATCC 12046 was a monomeric enzyme with an apparent molecular mass of 95 kDa, which is consistent with the monomeric PepN described for *Lb. helveticus* SBT 2171 [8] and CNRZ 32 [7]. Additionally, a trimeric form of an aminopeptidase with a molecular mass of approx. 129 kDa (∼43 kDa per subunit) was described for *Lb. helveticus* JCM 1004 [30].

#### Characterization of PepX

The optimal pH for PepX from *Lb. helveticus* ATCC 12046 was 6.5, which is identical to that reported for PepX from *Lb. helveticus* LHE-511 [12]. However, the PepX from *Lb. helveticus* ITG LH1 and CNRZ 32 demonstrated a slightly higher pH optimum of 7.0 [5], [11]. The temperature optimum of PepX from *Lb. helveticus* ATCC 12046 was determined to be 50°C, which is similar to that of the PepX from *Lb. helveticus* LHE-511 [12]. A lower optimal temperature of 40°C was measured for the PepX enzymes isolated from *Lb. helveticus* ITG LH1 [5] and CNRZ 32 [11]. The PepX from *Lb. helveticus* ATCC 12046 demonstrated a residual activity of 34% after incubation for 15 d at 50°C. In comparison, the PepX from *Lb. helveticus* ITG LH1 lost approx. 40% of its activity after 30 minutes at 50°C [5].

The kinetic parameters of PepX from *Lb. helveticus* ATCC 12046 were determined using three chromogenic peptides (H-Ala-Pro-*p*NA, H-Arg-Pro-*p*NA and H-Gly-Pro-*p*NA). The *K_m_* values ranged between 1.3 and 11.3 mM. These values were approx. 20-fold higher than those reported for the PepX from *Lb. helveticus* ITG LH1 [5]. The relative activity of PepX from *Lb. helveticus* ATCC 12046 for the three different substrates was determined in the order: H-Ala-Pro-*p*NA>H-Arg-Pro-*p*NA>H-Gly-Pro-*p*NA. This result is consistent with the results presented by Degraeve and Martial-Gros [5], as well as by Miyakawa *et al*. [12] for PepX from other *Lb. helveticus* strains. The exopeptidase PepX of the current study was product inhibited by some dipeptides that contained aromatic amino acids. This type of inhibition has not been described for other exopeptidases. However, some commercial preparations also demonstrated product inhibition by endopeptidases during food protein hydrolysis [13], [14], [31].

The influence of various mono- and divalent cations on the PepX activity of *Lb. helveticus* ATCC 12046 was investigated. A slight activating effect of NaCl and KCl was also observed for PepX from *Lb. helveticus* ITG LH1 [5] and CNRZ 32 [11]. The PepX from *Lb. helveticus* LHE-511 was not inhibited by 1 mM FeCl_2_ [12], whereas the PepX from *Lb. helveticus* ITG LH1 [5] and the PepX in this study were strongly inhibited. In addition, the PepX activity determined in this study and from *Lb. helveticus* LHE-511 [12] increased in the presence of 1 mM ZnCl_2_ to 137 and 111%, respectively. At an identical ZnCl_2_ concentration, the PepX activity from *Lb. helveticus* ITG LH1 [5] and CNRZ 32 [11] decreased to approx. 8 and 40% residual activity, respectively.

In our studies, PepX was inhibited by PMSF and was most likely a serine peptidase. This finding is consistent with previous reports. Residual activities of 66, 38, 34, and 46% were determined in the presence of 1 mM PMSF for PepX from the strains *Lb. helveticus* ATCC 12046 (the current study), ITG LH1 [5], LHE-511 [12], and CNRZ 32 [11], respectively. These results suggest the involvement of a serine residue in catalysis. Moreover, the PepX from *Lactococcus lactis* was described as a serine peptidase [32].

The reducing agents DTT and *β*-mercaptoethanol did not demonstrate an inactivating effect on PepX from *Lb. helveticus* ATCC 12046, which is similar to the results described for PepX from *Lb. helveticus* ITG LH1 [5] and LHE-511 [12].

The PepX from *Lb. helveticus* LHE-511 [12] and CNRZ 32 [11] were inhibited in the presence of metal-complexing agents, such as EDTA and 1,10-phenanthroline, thus indicating a metal ion dependency. This dependency was not observed in the current study or with the PepX from *Lb. helveticus* ITG LH1 [5]. In conclusion, the PepX described currently possesses clearly unique features and shares some characteristics of PepX peptidases described previously. Product inhibition has been demonstrated thus far only for the PepX described in this report.

#### Characterization of PepN

The PepN from *Lb. helveticus* ATCC 12046 demonstrated an identical pH optimum at 6.5 to the PepN from CNRZ 32 [7] and ITGL1 [33]. The PepN from *Lb. helveticus* LHE-511 [10] and JCM 1004 [30] exhibited a maximal activity at pH 7.0.

The PepN from *Lb. helveticus* ATCC 12046 demonstrated the highest activity at 30°C. Other PepNs demonstrated higher temperature optima: 37°C for the *Lb. helveticus* strain LHE-511 [10], 40°C for the JCM 1004 strain [30] and 45°C for the CNRZ 32 strain [7]. It was surprising that PepN from *Lb. helveticus* ATCC 12046 exhibited remarkable thermal stability, demonstrating 20% residual activity after 15 d of incubation at 50°C. In comparison, the PepN from *Lb. helveticus* JCM 1004 demonstrated only 5% residual activity after only 30 min of incubation at 50°C [30]. A similar weak thermal stability of approx. 30% residual activity was reported for the PepN from *Lb. helveticus* LHE-511 after 2 h of incubation at 50°C [10].

The kinetic parameters of PepN from *Lb. helveticus* ATCC 12046 were determined using nine commercially available chromogenic peptides. The relative activities measured demonstrated an identical order (H-Lys-*p*NA>H-Arg-*p*NA>H-Leu-*p*NA>H-Ala-*p*NA) to other PepN peptidases from *Lb. helveticus* LHE-511 [10], CNRZ 32 [7] and JCM 1004 [30]. It was remarkable that PepN from *Lb. helveticus* ATCC 12046 was able to hydrolyze H-Pro-*p*NA, which has only been reported for the PepN of *Lb. helveticus* SBT 2171 [8] thus far. Other PepNs (from *Lb. helveticus* LHE-511 [10], CNRZ 32 [7] and *Lb. curvatus* DPC2024 [34]) could not hydrolyze this substrate.

The substrate inhibition observed for the PepN from *Lb. helveticus* ATCC 12046 is reported for the first time. This information is valuable for potential biotechnological applications of the enzyme in the future, for example, in fed-batch biotransformation processes avoiding high substrate concentrations.

The addition of NaCl or KCl displayed a negative effect on the PepN activity in this study. These results are consistent with results for PepN from *Lb. helveticus* CNRZ 32 published previously [7]. The activity of PepN from *Lb. helveticus* ATCC 12046 and PepN from *Lb. helveticus* JCM 1004 [30] was reduced after the addition of 1 mM FeCl_2_, to 6 and 45.7% residual activity, respectively. A similar result (19% residual activity) was reported for PepN from *Lb. helveticus* LHE-511 [10]. Additionally, Cu^2+^ ions (1 mM) decreased the PepN activity of this study, *Lb. helveticus* JCM 1004 [30], *Lb. helveticus* CNRZ 32 [7], and *Lb. helveticus* LHE-511 [10] to 2, 71.7, 8, and 4% residual activity, respectively. The heavy metal salt HgCl_2_ (0.001 mM) strongly inactivated PepN (2% residual activity). The PepN from *Lb. helveticus* CNRZ 32 demonstrated more tolerance (10% residual activity at 100-fold higher 0.1 mM HgCl_2_) [7]. The addition of CoCl_2_ (1 mM) increased the activity of PepN (161%) in this study, as similarly reported for the PepN from *Lb. helveticus* JCM 1004 [30], CNRZ 32 [7] and LHE-511 [10], for which the activation reached 151, 179 and 181%, respectively. The most efficient reactivation of apo-PepN was observed using 0.25 mM CoCl_2_ (180% activity) in this study. Moreover, the activity of apo-PepN from *Lb. helveticus* SBT 2171 was restored up to 168% using 100 µM CoCl_2_ [8].

The strong inhibition of PepN in this study by metal-complexing reagents, such as EDTA and 1,10-phenanthroline, indicated that the enzyme is a metallopeptidase, as also described for other PepNs [7], [10].

#### Improved casein hydrolysis

The aminopeptidases PepX and PepN were applied to a prehydrolyzed casein solution because there are no endopeptidases that alone provide a high degree of protein hydrolysis [35]. The combined use of PepX and PepN increased the rDH by a remarkable 132% due to the complementary substrate specificities of both peptidases. The general potential of PepX combined with PepN from *Lactococcus lactis* ssp. *cremoris* AM2 in the enhancement of the enzymatic degradation of chemically synthesized peptides has been demonstrated [36]. The debittering effect of a tryptic digest of *β*-casein with porcine kidney general aminopeptidase and X-prolyl dipeptidyl aminopeptidase from *Lc. lactis* ssp. *cremoris* AM2 was described [37].

A limited productivity of the peptidases is assumed in enzymatic protein hydrolysis using a batch process due to potential substrate or product inhibition. These types of inhibition were recognized for PepX and PepN in our study. Consequently, a continuous process, through the use of an enzymatic membrane reactor (EMR), would overcome these limitations, as was recently demonstrated in the casein hydrolysis using the commercial peptidase preparation BLAP (*Bacillus lentus* alkaline peptidases) [31], whereby the inhibiting peptides produced were continuously separated from the enzymes by ultrafiltration membranes. In such a system, stable enzymes, such as the PepX and PepN described, might be used for a long operating time at low substrate and product concentrations. In addition, a standardized product quality of the protein hydrolysate with no bitter taste and no allergenic effects would be possible in the future.

### Conclusion

Recombinant production of the exopeptidases PepX and PepN from *Lb. helveticus* ATCC 12046 in *E. coli* resulted in high levels of soluble enzymes. Both peptidases were easily purified using His-tag affinity chromatography (IMAC) and featured a reasonable thermostability at 50°C for 15 d. The kinetic investigations of both purified peptidases demonstrated product inhibition. Substrate inhibition was also observed for PepN. PepN possessed a broad substrate specificity in which even H-Pro-*p*NA was hydrolyzed. The synergistic effect of PepX and PepN resulted in a significantly higher degree of casein hydrolysis when used in combination. The properties of both enzymes have the potential for future applications in food protein hydrolysis using an enzyme membrane reactor.

## Supporting Information

Figure S1 **Purification chromatogram of PepX.** This file contains the chromatogram of the automated purification of PepX.
(PDF)

Figure S2 **SDS- and native PAGE.** This file contains the SDS and native PAGE analyses for recombinant PepX and PepN during purification.
(PDF)

Sequence S1 **Sequences of** ***pepX*** **/PepX.** This file contains the nucleotide sequence of the *pepX* gene and the translated amino acid sequence from *Lactobacillus helveticus* ATCC 12046.
(PDF)

Sequence S2 **Sequences of** ***pepN*** **/PepN.** This file contains the nucleotide sequence of the *pepN* gene and the translated amino acid sequence from *Lactobacillus helveticus* ATCC 12046.
(PDF)
