# Supplementary material for: Characterization of the Recombinant Exopeptidases PepX and PepN from Lactobacillus helveticus ATCC 12046 Important for Food Protein Hydrolysis
Source: PLoS One. 2013 Jul 19;8(7):e70055. doi: 10.1371/journal.pone.0070055 (PMC3716637; doi:10.1371/journal.pone.0070055)

Figure S1 Automated purification of recombinant PepX using Ni^{2+}-affinity chromatography (A) and desalting (B). The purified and desalted PepX is hatched.

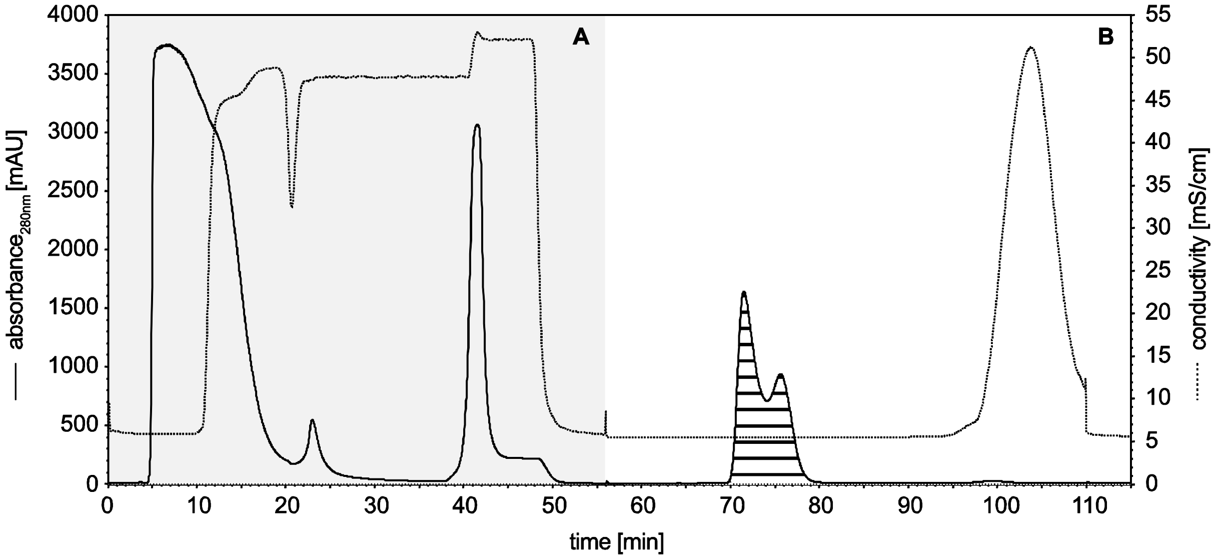

Supplement: Figure S1 — Purification chromatogram of PepX. This file contains the chromatogram of the automated purification of PepX. (PDF) [file pone.0070055.s001.pdf]
