# Supplementary material for: Characterization of the Recombinant Exopeptidases PepX and PepN from Lactobacillus helveticus ATCC 12046 Important for Food Protein Hydrolysis
Source: PLoS One. 2013 Jul 19;8(7):e70055. doi: 10.1371/journal.pone.0070055 (PMC3716637; doi:10.1371/journal.pone.0070055)

Figure S2 SDS-PAGE (A: PepX; B: PepN) and native-PAGE (C) of recombinant PepX and PepN produced in *E. coli* BL21 (DE3) following purification.

SDS-PAGEs: M: Molecular weight marker; lane 1: crude cell extract before purification; lane 2: pooled flow-through fractions: lane 3: pooled purified fractions after desalting; lanes 1 – 3: 5 µg protein each.

Native-PAGE: M: Molecular weight marker; lane 1 and 3: purified PepX; lane 2 and 4: purified PepN; lanes 1 – 4: 5 µg protein each

The SDS-PAGE and the marker of the native-PAGE were Coomassie stained; lanes 3 and 4 of the native-PAGE were active stained.

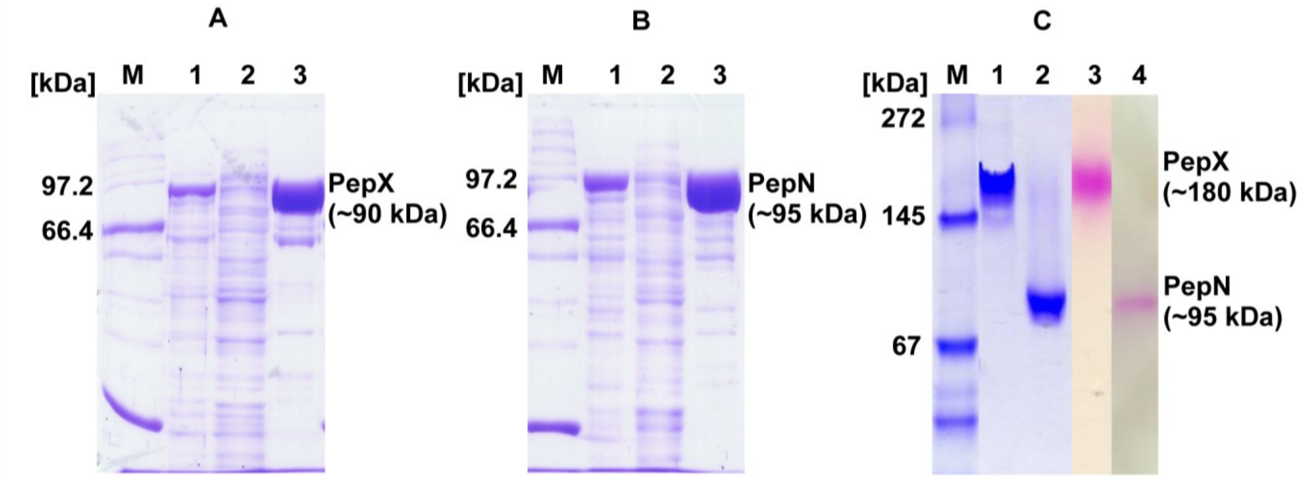

Supplement: Figure S2 — SDS- and native PAGE. This file contains the SDS and native PAGE analyses for recombinant PepX and PepN during purification. (PDF) [file pone.0070055.s002.pdf]
