# Supplementary material for: Characterization of the Recombinant Exopeptidases PepX and PepN from Lactobacillus helveticus ATCC 12046 Important for Food Protein Hydrolysis
Source: PLoS One. 2013 Jul 19;8(7):e70055. doi: 10.1371/journal.pone.0070055 (PMC3716637; doi:10.1371/journal.pone.0070055)
Supplement: Sequence S1 — Sequences of pepX /PepX. This file contains the nucleotide sequence of the pepX gene and the translated amino acid sequence from Lactobacillus helveticus ATCC 12046. (PDF) [file pone.0070055.s003.pdf]

Sequence S1 Nucleotide sequence of the *pepX* gene and the translated amino acid sequence from *Lactobacillus helveticus* ATCC 12046. Elements from the pET20b (+) are underlined. The restriction sites (*NdeI* and *XhoI*) are noted in *italics*. The last amino acid of PepX is K. The amino acids L and E originate from the *XhoI* restriction site, followed by the His$_6$ tag. The stop codon is denoted by *.

```
                NdeI
GGA GAT ATA CAT ATG AAA TAT AAC CAA TAT GCT TAC GTT GAA ACC GAC TTT CAA CAA CAA  < 60
                M   K   Y   N   Q   Y   A   Y   V   E   T   D   F   Q   Q   Q

GTT AAA GAA TTA ATC GAT ATC AAC TTC TTA CCT AAA AAT TAT CAA GTC TGG GAC TTC AGC  < 120
V   K   E   L   I   D   I   N   F   L   P   K   N   Y   Q   V   W   D   F   S

AGT CTT TTA GCT AAA TTA GTT AAA AAT GCA ATC GCT GAG GCT AAA ACA GAT GCC GCT AAA  < 180
S   L   L   A   K   L   V   K   N   A   I   A   E   A   K   T   D   A   A   K

AAT GCC AAA CTT GCG GAA TTT GCA GTT TCT GAT CAC CAG ACC TTG GCT GAT TTT CTT AAG  < 240
N   A   K   L   A   E   F   A   V   S   D   H   Q   T   L   A   D   F   L   K

GAA AAG CCA ACA GAA ATT GGT ACC AAA CAA TTC TAC AAC GTA GCT CTG CAA CTT CTT GGC  < 300
E   K   P   T   E   I   G   T   K   Q   F   Y   N   V   A   L   Q   L   L   G

TAC CAC GTC CAC TAC GAC TAT GAT TTT GCT GAT CCA ACC GGT TTC ATG CAA AGA AAT GCT  < 360
Y   H   V   H   Y   D   Y   D   F   A   D   P   T   G   F   M   Q   R   N   A

TTG CCA TTT TTG CAA GAC ATC AGC GAC AAT CAA AAA TTA ATC TCT GCC TTT TAC CGC TTG  < 420
L   P   F   L   Q   D   I   S   D   N   Q   K   L   I   S   A   F   Y   R   L

CTC AAT ACC CGT GCT AAA AAC GGA CAA ATC TTG CTC GAT GTG ATG GCA GGC AAA GGC TAC  < 480
L   N   T   R   A   K   N   G   Q   I   L   L   D   V   M   A   G   K   G   Y

TTT ACT CAA TTC TGG GGT CAA AAC AAG TTC AAG TTC TTC AAT GGT AAA TCT ATC CCT GTT  < 540
F   T   Q   F   W   G   Q   N   K   F   K   F   F   N   G   K   S   I   P   V

TTT GAC ACA AAT AAG GTC ATT CGT GAA GTC GTT TAT GTC GAA ACT GAT CTT GAT ACT GAC  < 600
F   D   T   N   K   V   I   R   E   V   V   Y   V   E   T   D   L   D   T   D

CAT GAT GGC AAG AGT GAT TTA ATT CAA GTT ACT GTT TTC CGT CCT GAA GAA ACT AAT AAG  < 660
H   D   G   K   S   D   L   I   Q   V   T   V   F   R   P   E   E   T   N   K

GGC CTT AAA GTA CCT GCA CTC TAC ACT GCA TCC CCA TAC TTT GGC GGA ATC ATC GCC AAC  < 720
G   L   K   V   P   A   L   Y   T   A   S   P   Y   F   G   G   I   I   A   N

GAA AAA CGC AAC CAT AAC GTT GAC GAA AAT CTC TCT GAC TCT ACC GAA TGG AAT GAT CCA  < 780
E   K   R   N   H   N   V   D   E   N   L   S   D   S   T   E   W   N   D   P

CAA TAC GTT CAC TCT CCA ATC GTT AAG GCT GAA AAG CCA GAT GGT TCA AGC CGT CCA GCT  < 840
Q   Y   V   H   S   P   I   V   K   A   E   K   P   D   G   S   S   R   P   A

ACA GAG GAG GCA GTG CAC AAG TCC TCT TAC CCA TTA AAC GAA TAC ATG CTA GCT CGC GGT  < 900
T   E   E   A   V   H   K   S   S   Y   P   L   N   E   Y   M   L   A   R   G

TTT GCT AGC GTC TTT GCT GGT GCA ATT GGT ACT CGC GGC AGC GAT GGT GTC CGC ATC ACT  < 960
F   A   S   V   F   A   G   A   I   G   T   R   G   S   D   G   V   R   I   T

GGT GCT CCA GAA GAA ACT GAA TCA GCT GCT GCA GTT ATC GAA TGG CTG CAT GGT GAT CGT  < 1020
G   A   P   E   E   T   E   S   A   A   A   V   I   E   W   L   H   G   D   R

GTT GCC TAC ACT GAT AGA ACC AGA ACC GTT CAA ACT ACA GCT GAT TGG TGC AAC GGC AAC  < 1080
V   A   Y   T   D   R   T   R   T   V   Q   T   T   A   D   W   C   N   G   N

ATC GGT ATG ACC GGT CGT TCA TAT CTT GGT ACT TTG CAA ATC GCC ATT GCC ACA ACT GGC  < 1140
I   G   M   T   G   R   S   Y   L   G   T   L   Q   I   A   I   A   T   T   G

GTT AAG GGT CTT AAG ACT GTC GTT TCT GAA GCC GCA ATT TCA TCA TGG TAC GAC TAC TAC  < 1200
V   K   G   L   K   T   V   V   S   E   A   A   I   S   S   W   Y   D   Y   Y

CGT GAA CAT GGT TTG GTT ATT GCT CCT GAA GCT TGC CAA GGT GAA GAC CTT GAT TTG CTC  < 1260
R   E   H   G   L   V   I   A   P   E   A   C   Q   G   E   D   L   D   L   L
```

```
GCT GAA ACC TGT CAA TCA AAC TTA TGG GAT GCC GGT TCA TAC CTT AAG ATC AAG CCT GAA  < 1320
A   E   T   C   Q   S   N   L   W   D   A   G   S   Y   L   K   I   K   P   E

TAC GAC AAA ATG CAA AAG CAG TTG CGT GAA AAA GAA GAC CGT AAT ACT GGT CAA TAC TCT  < 1380
Y   D   K   M   Q   K   Q   L   R   E   K   E   D   R   N   T   G   Q   Y   S

GAC TTC TGG GAA GCT AGA AAC TAC CGT CAC CAC GCT GAT GGT ATC AAG TGC TCC TGG ATT  < 1440
D   F   W   E   A   R   N   Y   R   H   H   A   D   G   I   K   C   S   W   I

TCC GTT CAT GGT TTA AAT GAC TGG AAT GTT AAG CCA AAG AAC GTT TAC AAA ATC TGG CAA  < 1500
S   V   H   G   L   N   D   W   N   V   K   P   K   N   V   Y   K   I   W   Q

CTA GTT AAA AAG ATG CCA ATG AAG CAT CAC CTC TTC TTG CAC CAA GGT CCG CAC TAC AAT  < 1560
L   V   K   K   M   P   M   K   H   H   L   F   L   H   Q   G   P   H   Y   N

ATG AAC AAC TTA GTT TCC ATC GAC TTT ACA GAC TTA ATG AAC CTC TGG TTT GTT CAT GAA  < 1620
M   N   N   L   V   S   I   D   F   T   D   L   M   N   L   W   F   V   H   E

CTT TTA GGC ATT GAA AAT AAT GCT TAC AAT CAA TGG CCA ACG GTT ATG ATT CAA GAT AAC  < 1680
L   L   G   I   E   N   N   A   Y   N   Q   W   P   T   V   M   I   Q   D   N

TTG CAA GCT GAC AAG TGG CAT GAA GAA CCT GAC TGG AGC AAT GAT TTA GGC CAA GAA AAG  < 1740
L   Q   A   D   K   W   H   E   E   P   D   W   S   N   D   L   G   Q   E   K

ATC TAC TAC CCT ACT GAT GAA GGC GAA CTT TTC CAA GAT GGC AAC GGC AAA GCA CAA AAG  < 1800
I   Y   Y   P   T   D   E   G   E   L   F   Q   D   G   N   G   K   A   Q   K

TCC TTT ACC GAT GTT GGC GGT ATT GAA TTC AAG AAA GCT GGT ATT TCA GAA AGC GAT TGG  < 1860
S   F   T   D   V   G   G   I   E   F   K   K   A   G   I   S   E   S   D   W

CAA TAC AAG TTC ATT TGC GGT GAT GAA AAA TGG GCT AAG CCA AGT TTA CGT TTT GAA ACT  < 1920
Q   Y   K   F   I   C   G   D   E   K   W   A   K   P   S   L   R   F   E   T

GAT GAA TTT ACC CAC CCT ACT ACC ATC GTG GGT CGT CCA GAA GTT AAA GTT AGA GTT TCT  < 1980
D   E   F   T   H   P   T   T   I   V   G   R   P   E   V   K   V   R   V   S

GCT AGT CTG CCA AAG GGT GAG ATT TCC GTT GCC TTA GTT GAA CTG GGC GAA AGA CAA CGA  < 2040
A   S   L   P   K   G   E   I   S   V   A   L   V   E   L   G   E   R   Q   R

CTT ACT GCT ACT CCT AAA TTC TTA ATG CAT GGC GGA CAA GAA TTA GGC TAC CGT TTT GGC  < 2100
L   T   A   T   P   K   F   L   M   H   G   G   Q   E   L   G   Y   R   F   G

ACT GAC ACT TTA CAA GAA TTT GTT CCA GAT AAA AAG ACT AAA GCC AAG TTG ATT ACT AAG  < 2160
T   D   T   L   Q   E   F   V   P   D   K   K   T   K   A   K   L   I   T   K

GCT CAC ATG AAC TTG CAA AAC TTC AAG GAT ATG AAG AAG CCT GAA GCA ATC GAC GCC GAT  < 2220
A   H   M   N   L   Q   N   F   K   D   M   K   K   P   E   A   I   D   A   D

AAG TTC TAC GAC TTG GAC TTC TTG CTT CAA CCT ACT TAT TAC ACC ATC CCA TCT GGC AGC  < 2280
K   F   Y   D   L   D   F   L   L   Q   P   T   Y   Y   T   I   P   S   G   S

AAG CTT GCT TTG ATC ATT TAT TCA ACT GAC CAA GGC ATG ACT AAG CGT CCG CTT GAA GAT  < 2340
K   L   A   L   I   I   Y   S   T   D   Q   G   M   T   K   R   P   L   E   D
                                                                 XhoI
GAA ACT TAC ACA ATT GAT TTA GCT AAT ACT GAA ATC AAG TTT TAT GAA AAA CTC GAG CAC  < 2400
E   T   Y   T   I   D   L   A   N   T   E   I   K   F   Y   E   K   L   E   H

CAC CAC CAC CAC CAC TGA GAT CCG GCT GCT AAC                                      < 2433
H   H   H   H   H   *
```
