# Supplementary material for: Characterization of the Recombinant Exopeptidases PepX and PepN from Lactobacillus helveticus ATCC 12046 Important for Food Protein Hydrolysis
Source: PLoS One. 2013 Jul 19;8(7):e70055. doi: 10.1371/journal.pone.0070055 (PMC3716637; doi:10.1371/journal.pone.0070055)
Supplement: Sequence S2 — Sequences of pepN /PepN. This file contains the nucleotide sequence of the pepN gene and the translated amino acid sequence from Lactobacillus helveticus ATCC 12046. (PDF) [file pone.0070055.s004.pdf]

**Sequence S2** Nucleotide sequence of the *pepN* gene and the translated amino acid sequence from *Lactobacillus helveticus* ATCC 12046. Elements from the pET20b (+) are underlined. The restriction sites (*NdeI* and *XhoI*) are noted in *italics*. The last amino acid of PepN is D. The amino acids L and E originate from the *XhoI* restriction site, followed by the $His_6$ tag. The stop codon is denoted by *.

```
            NdeI
GGA GAT ATA CAT ATG GCA GTT AAA CGT TTC TAT AAA ACT TTC CAC CCA GAA CAT TAC GAT  < 60
                M   A   V   K   R   F   Y   K   T   F   H   P   E   H   Y   D

TTG CGT ATT AAT GTA AAC CGT AAG AAT AAA ACT ATT AAT GGT ACT TCC ACA ATT ACT GGT  < 120
L   R   I   N   V   N   R   K   N   K   T   I   N   G   T   S   T   I   T   G

GAT GTA ATT GAA AAT CCA GTA TTT ATT AAC CAA AAA TTT ATG ACC ATT GAT AGC GTT AAG  < 180
D   V   I   E   N   P   V   F   I   N   Q   K   F   M   T   I   D   S   V   K

GTT GAT GGT AAA AAT GTT GAT TTT GAT GTA ATC GAA AAA GAT GAA GCA ATC AAA ATT AAA  < 240
V   D   G   K   N   V   D   F   D   V   I   E   K   D   E   A   I   K   I   K

ACA GGA GTA ACT GGC AAA GCT GTA ATC GAA ATT GCT TAT AGC GCA CCA CTT ACT GAT ACT  < 300
T   G   V   T   G   K   A   V   I   E   I   A   Y   S   A   P   L   T   D   T

ATG ATG GGT ATT TAT CCT TCA TAT TAC GAA TTA GAA GGT AAG AAG AAG CAA ATC ATC GGT  < 360
M   M   G   I   Y   P   S   Y   Y   E   L   E   G   K   K   K   Q   I   I   G

ACG CAA TTC GAA ACT ACT TTT GCT CGC CAA GCA TTC CCA TGT GTG GAC GAA CCT GAA GCT  < 420
T   Q   F   E   T   T   F   A   R   Q   A   F   P   C   V   D   E   P   E   A

AAA GCT ACA TTC TCA CTT GCT CTT AAG TGG GAT GAA CAA GAT GGT GAA GTT GCA CTT GCT  < 480
K   A   T   F   S   L   A   L   K   W   D   E   Q   D   G   E   V   A   L   A

AAT ATG CCA GAA GTA GAA GTT GAC AAG GAT GGC TAC CAC CAC TTT GAA GAA ACT GTC CGC  < 540
N   M   P   E   V   E   V   D   K   D   G   Y   H   H   F   E   E   T   V   R

ATG TCT AGT TAC TTA GTT GCC TTT GCC TTT GGT GAA TTA CAA TCT AAG ACT ACT CAT ACT  < 600
M   S   S   Y   L   V   A   F   A   F   G   E   L   Q   S   K   T   T   H   T

AAG GAT GGC GTA TTA ATT GGC GTT TAC GCA ACT AAG GCA CAC AAG CCT AAG GAA TTA GAC  < 660
K   D   G   V   L   I   G   V   Y   A   T   K   A   H   K   P   K   E   L   D

TTC GCT TTG GAC ATT GCT AAG CGC GCA ATT GAA TTT TAC GAA GAA TTC TAC CAA ACC AAA  < 720
F   A   L   D   I   A   K   R   A   I   E   F   Y   E   E   F   Y   Q   T   K

TAC CCA CTT CCA CAG TCA TTG CAA CTT GCA TTG CCA GAC TTC TCA GCT GGT GCC ATG GAA  < 780
Y   P   L   P   Q   S   L   Q   L   A   L   P   D   F   S   A   G   A   M   E

AAC TGG GGT CTT GTA ACT TAC CGT GAA GCA TAC TTG CTC CTT GAC CCA GAC AAT ACT AGC  < 840
N   W   G   L   V   T   Y   R   E   A   Y   L   L   L   D   P   D   N   T   S

TTG GAA ATG AAG AAG TTA GTT GCC ACA GTT ATT ACC CAT GAA TTG GCT CAC CAA TGG TTC  < 900
L   E   M   K   K   L   V   A   T   V   I   T   H   E   L   A   H   Q   W   F

GGT GAC TTG GTA ACC ATG AAG TGG TGG GAC AAC TTA TGG CTT AAC GAA AGT TTT GCC AAC  < 960
G   D   L   V   T   M   K   W   W   D   N   L   W   L   N   E   S   F   A   N

ATG ATG GAA TAC TTA TCA GTT GAT GGT TTG GAA CCT GAC TGG CAC ATC TGG GAA ATG TTC  < 1020
M   M   E   Y   L   S   V   D   G   L   E   P   D   W   H   I   W   E   M   F

CAA ACT AGT GAA GCT GCA TCT GCA TTG AAC AGA GAT GCT ACT GAT GGT GTT CAA CCA ATT  < 1080
Q   T   S   E   A   A   S   A   L   N   R   D   A   T   D   G   V   Q   P   I

CAA ATG GAA ATT AAT GAC CCA GCA GAT ATT GAC TCA GTC TTT GAC GGT GCC ATT GTT TAT  < 1140
Q   M   E   I   N   D   P   A   D   I   D   S   V   F   D   G   A   I   V   Y

GCT AAG GGT TCA AGA ATG TTA GTC ATG GTT CGT TCA CTT CTT GGT GAC GAC GCA CTT CGC  < 1200
A   K   G   S   R   M   L   V   M   V   R   S   L   L   G   D   D   A   L   R

AAG GGT CTT AAG TAC TAC TTC GAC CAC CAC AAG TTT GGC AAT GCC ACT GGT GAT GAC CTT  < 1260
K   G   L   K   Y   Y   F   D   H   H   K   F   G   N   A   T   G   D   D   L
```

```
TGG GAT GCA CTT TCA ACT GCA ACT GAT CTT GAT ATT GGT AAA ATC ATG CAC TCA TGG CTT  < 1320
W   D   A   L   S   T   A   T   D   L   D   I   G   K   I   M   H   S   W   L

AAG CAA CCT GGT TAC CCA GTA GTT AAT GCC TTC GTT GCT GAA GAT GGT CAT TTG AAG CTT  < 1380
K   Q   P   G   Y   P   V   V   N   A   F   V   A   E   D   G   H   L   K   L

ACG CAA AAG CAA TTC TTC ATT GGT GAA GGT GAA GAT AAA GGT AGA CAA TGG CAA ATT CCA  < 1440
T   Q   K   Q   F   F   I   G   E   G   E   D   K   G   R   Q   W   Q   I   P

TTG AAT GCT AAC TTC GAC GCA CCT AAG ATT ATG TCT GAT AAG GAA ATT GAC TTA GGC AAT  < 1500
L   N   A   N   F   D   A   P   K   I   M   S   D   K   E   I   D   L   G   N

TAC AAG GTA CTT CGC GAA GAA GCA GGT CAC CCA CTT AGA CTT AAT GTT GGC AAC AAC TCA  < 1560
Y   K   V   L   R   E   E   A   G   H   P   L   R   L   N   V   G   N   N   S

CAC TTC ATC GTT GAA TAT GAC AAG ACT TTG CTT GAT GAT ATT TTG TCA GAT GTT AAT GAA  < 1620
H   F   I   V   E   Y   D   K   T   L   L   D   D   I   L   S   D   V   N   E

TTG GAT CCA ATT GAT AAG TTA CAA TTG CTT CAA GAC TTA CGT CTC TTA GCA GAA GGT AAG  < 1680
L   D   P   I   D   K   L   Q   L   L   Q   D   L   R   L   L   A   E   G   K

CAA ATT TCT TAC GCT TCA ATT GTT CCA CTT TTG GTT AAA TTC GCT GAT TCT AAG TCA AGC  < 1740
Q   I   S   Y   A   S   I   V   P   L   L   V   K   F   A   D   S   K   S   S

TTG GTA ATT AAC GCA CTT TAC ACT ACT GCA GCT AAG CTT CGT CAA TTC GTT GAA CCA GAA  < 1800
L   V   I   N   A   L   Y   T   T   A   A   K   L   R   Q   F   V   E   P   E

TCA AAT GAA GAA AAG AAC TTG AAG AAG CTT TAT GAT CTC TTA TCA AAG GAT CAA GTT GCA  < 1860
S   N   E   E   K   N   L   K   K   L   Y   D   L   L   S   K   D   Q   V   A

CGT TTA GGC TGG GAA GTA AAG CCA GGC GAA AGC GAT GAA GAT GTT CAA ATT CGT CCA TAC  < 1920
R   L   G   W   E   V   K   P   G   E   S   D   E   D   V   Q   I   R   P   Y

GAG TTG AGC GCA AGT CTT TAC GCT GAA AAT GCG GAC TCA ATT AAG GCA GCT CAC CAA ATC  < 1980
E   L   S   A   S   L   Y   A   E   N   A   D   S   I   K   A   A   H   Q   I

TTT ACT GAA AAT GAA GAT AAC TTG GAA GCA TTG AAT GCA GAT ATT CGT CCA TAC GTT TTA  < 2040
F   T   E   N   E   D   N   L   E   A   L   N   A   D   I   R   P   Y   V   L

ATC AAT GAA GTT AAG AAC TTT GGC AAT GCT GAA TTA GTT GAT AAG TTA ATT AAG GAA TAC  < 2100
I   N   E   V   K   N   F   G   N   A   E   L   V   D   K   L   I   K   E   Y

CAA AGA ACA GCT GAC CCA TCA TAC AAG GTT GAC TTA CGC AGC GCT GTA ACC AGC ACC AAG  < 2160
Q   R   T   A   D   P   S   Y   K   V   D   L   R   S   A   V   T   S   T   K

GAT CTT GCA GCT ATC AAG GCT ATT GTT GGC GAC TTT GAA AAT GCT GAC GTA GTT AAG CCA  < 2220
D   L   A   A   I   K   A   I   V   G   D   F   E   N   A   D   V   V   K   P

CAA GAT TTA TGT GAT TGG TAC CGT GGT TTA CTT GCT AAC CAT TAT GGT CAA CAA GCA GCT  < 2280
Q   D   L   C   D   W   Y   R   G   L   L   A   N   H   Y   G   Q   Q   A   A

TGG GAC TGG ATC AGA GAA GAC TGG GAT TGG CTT GAC AAG ACT GTT GGT GGT GAC ATG GAA  < 2340
W   D   W   I   R   E   D   W   D   W   L   D   K   T   V   G   G   D   M   E

TTT GCT AAA TTT ATC ACT GTT ACT GCA GGC GTC TTC CAT ACC CCA GAA AGA CTT AAG GAA  < 2400
F   A   K   F   I   T   V   T   A   G   V   F   H   T   P   E   R   L   K   E

TTC AAA GAA TTC TTT GAA CCA AAG ATT AAT GTT CCA CTT CTT AGT CGT GAA ATT AAG ATG  < 2460
F   K   E   F   F   E   P   K   I   N   V   P   L   L   S   R   E   I   K   M

GAC GTT AAG GTC ATC GAA AGC AAG GTT AAC TTG ATC GAA GCT GAA AAA GAT GCT GTT AAT  < 2520
D   V   K   V   I   E   S   K   V   N   L   I   E   A   E   K   D   A   V   N
                                    XhoI
GAT GCA GTT GCT AAA GCA ATT GAT CTC GAG CAC CAC CAC CAC CAC CAC TGA GAT CCG GCT  < 2580
D   A   V   A   K   A   I   D   L   E   H   H   H   H   H   H   *

GCT AAC                                                                          < 2587
```
